# Recording Saltatory Conduction Along Sensory Axons Using a High-Density Microelectrode Array

**DOI:** 10.3389/fnins.2022.854637

**Published:** 2022-04-18

**Authors:** Kenta Shimba, Takahiro Asahina, Koji Sakai, Kiyoshi Kotani, Yasuhiko Jimbo

**Affiliations:** ^1^Department of Precision Engineering, School of Engineering, The University of Tokyo, Tokyo, Japan; ^2^Japan Society for Promotion of Science, Tokyo, Japan; ^3^Research Center for Advanced Science and Technology, The University of Tokyo, Tokyo, Japan

**Keywords:** saltatory conduction, high density microelectrode array, myelination, sensory neuron, axon

## Abstract

Myelinated fibers are specialized neurological structures used for conducting action potentials quickly and reliably, thus assisting neural functions. Although demyelination leads to serious functional impairments, little is known the relationship between myelin structural change and increase in conduction velocity during myelination and demyelination processes. There are no appropriate methods for the long-term evaluation of spatial characteristics of saltatory conduction along myelinated axons. Herein, we aimed to detect saltatory conduction from the peripheral nervous system neurons using a high-density microelectrode array. Rat sensory neurons and intrinsic Schwann cells were cultured. Immunofluorescence and ultrastructure examination showed that the myelinating Schwann cells appeared at 1 month, and compact myelin was formed by 10 weeks *in vitro*. Activity of rat sensory neurons was evoked with optogenetic stimulation, and axon conduction was detected with high-density microelectrode arrays. Some conductions included high-speed segments with low signal amplitude. The same segment could be detected with electrical recording and immunofluorescent imaging for a myelin-related protein. The spatiotemporal analysis showed that some segments show a velocity of more than 2 m/s and that ends of the segments show a higher electrical sink, suggesting that saltatory conduction occurred in myelinated axons. Moreover, mathematical modeling supported that the recorded signal was in the appropriate range for axon and electrode sizes. Overall, our method could be a feasible tool for evaluating spatial characteristics of axon conduction including saltatory conduction, which is applicable for studying demyelination and remyelination.

## Introduction

Myelinated nerve fibers are important structures that transfer action potentials quickly and reliably, maintaining neural functioning. The myelin sheath formation on axons is known to increase conduction velocity and improve energy efficiency by reducing leakage and clustering Na channels (Nave, 2010; Aggarwal et al., 2011), a process known as saltatory conduction. The relationship between myelination and conduction velocity is under debate, as some reports have suggested that the formation of clusters of Na channels is sufficient to increase conduction velocity (Neishabouri and Faisal, 2014; Bonetto et al., 2019). Additionally, the microstructure of the myelin sheath may contribute to conduction velocity regulation, and myelinating glial cells can change these microstructures to respond to synaptic plasticity (Cullen et al., 2021), suggesting that myelinating glial cells are actively involved in the regulation of computational functions in the nervous system. Myelin sheath formation and microstructure changes occur in timeframes of hours and days. Therefore, long-term direct measurements of saltatory conduction in myelinated fibers are needed to elucidate changes in conduction velocity during myelination, glial regulation, and subsequent network changes.

Myelination-regulated axonal conduction has been studied using patch-clamp techniques and voltage-sensitive dyes (VSD). Traditionally, the conduction time has been measured between two points on the nerve (Raastad and Shepherd, 2003; Hu and Shu, 2012). A new method directly applies a pipette to the node of Ranvier, which can determine the specific expression profiles of ion channels (Kanda et al., 2019). The importance of the peri-axonal space between the myelin sheath and axon was revealed by comparing data obtained using voltage-sensitive dyes and a mathematical model (Cohen et al., 2020). The patch-clamp method has high temporal resolution and signal-to-noise ratio but can only measure the potential at limited locations. VSD imaging has high spatial resolution but limited signal-to-noise ratio. Additionally, both methods are invasive and therefore not suitable for long-term measurements.

Reports on the use of microelectrode arrays (MEAs) for evaluation of the shape and conduction velocity of axons over an extended period are increasing (Bakkum et al., 2013; Lewandowska et al., 2015; Shimba et al., 2016; Radivojevic et al., 2017; Bullmann et al., 2019; Yuan et al., 2020); these are less invasive and suitable for long-term measurement. Changes in conduction velocity in unmyelinated axons caused by repetitive firing spontaneously occur within several seconds (Shimba et al., 2015). With its high spatiotemporal resolution, the initial segment of an axon is the major contributor toward extracellular electrical potential landscapes (Bakkum et al., 2018). However, detection of saltatory conduction in myelinated fibers through electrical recordings using MEAs is unreported.

Here, we aimed to electrically detect saltatory conduction from neurons of the peripheral nervous system using high-density MEA (HD-MEA). Rat dorsal root ganglion (DRG) cells were cultured, and myelin sheaths were produced on the axons of DRG neurons by endogenous Schwann cells. In separate experiments, as a part of this study, the formation of the myelin sheath was examined using immunofluorescent staining and transmission electron microscopy (TEM). The spatiotemporal conduction characteristics were evaluated by identifying axons using HD-MEA. Finally, we compared the spatial and temporal properties of extracellular potential between recorded data and a mathematical model in which the parameters included those observed under a microscope.

## Materials and Methods

### Cell Culture

Maxone HD-MEA chips (Maxwell Biosystems AG, Zurich, Switzerland) were pretreated and coated according to the protocol of Maxwell Biosystems. The chips were immersed in 70% ethanol for 30 min after hydrophilization. After rinsing with ultrapure water and drying them, 0.07% polyethyleneimine (Sigma-Aldrich Co., Ltd., St. Louis, MO, United States) was added around electrodes, and the chips were left at 4°C overnight. After rinsing four times with sterile water, 20 μg/mL laminin (Thermo Fisher Scientific, Waltham, MA, United States) was added and incubated at 37°C for ≥ 1 h.

All procedures were performed according to the University of Tokyo Animal Experiment Manual with the approval of the University of Tokyo Animal Experiment Committee (KA19-14). DRGs were dissected out from 15-day-old Wistar rat embryos (Charles River Laboratory Japan Inc., Kanagawa, Japan) and enzymatically treated using 0.25% Trypsin diluted in Hanks’ balanced salts solution (FUJIFILM Wako Pure Chemical Corp., Osaka, Japan) at 37°C for 17 min. The cells were isolated by pipetting and seeded on HD-MEA at an initial density of 500 cells/mm^2^ after laminin removal. We used a neurobasal medium supplemented with B27 supplement (Thermo Fisher Scientific), 1% penicillin–streptomycin (Thermo Fisher Scientific), 4% w/v D-glucose (Sigma-Aldrich), and 50 ng/mL nerve growth factor (FUJIFILM Wako Pure Chemical Corp.), as previously described (Sakai et al., 2018). From day 6, myelin formation was induced by the addition of 50 μg/mL ascorbic acid (Sigma-Aldrich). Adeno-associated virus (AAV) particles [pAAV-Syn-ChR2 (H134R)-GFP; gift from Edward Boyden, Massachusetts Institute of Technology, MA, United States; Addgene #58880-AAV8] were added to DRG neurons at 20 days *in vitro* (DIV) for expressing channel rhodopsin 2 (ChR2) and green fluorescent protein (GFP). DRG neurons were transduced with 5.0 × 10^9^ genome copies of AAVs in 0.8 mL of media per chip. The culture medium was changed twice a week. The samples were placed in a humidified 5% CO_2_ incubator at 37°C.

### Activity Measurement

The MaxOne recording system (Maxwell Biosystems) was used to measure the activity of DRG neurons. An HD-MEA chip has 26,400 electrodes and can switch the circuit and record extracellular potential from 1,020 electrodes simultaneously. We stimulated DRG neurons expressing ChR2 protein to induce activity because DRG neurons rarely show spontaneous activity. The fluorescence of GFP was observed with a fluorescence microscope, and GFP-positive neurons were manually selected. Each neuron was stimulated with 50 μm × 50 μm square light pulses of 5 ms duration at intervals of 2 s. Optogenetic stimulation was performed with Polygon 1000-G Pattern Illuminator system (Mightex Systems, CA, United States).

To record axonal activity, we used MaxLab Live (Maxwell Biosystems) and pre-defined recording pipelines as shown previously (Bakkum et al., 2013). We first measured all electrodes while scanning each for 60 s using the “activity scan” mode (Supplementary Figure 1A). We then selected the electrodes that recorded signals from cell bodies based on the scan results (Supplementary Figures 1B,C). The cell body electrodes were fixed, and the signals from the other electrodes were recorded by running scans (Supplementary Figure 1D). In total, 28–33 sets of recordings were made per sample, with each set lasting for 90 s. The signals were recorded at 10 bits and 20 kHz before filtering. The samples were placed in a custom-made recording chamber at 37°C. Activity recording was performed at 35 DIV with three samples and at 9 weeks *in vitro* (WIV; 58–64 DIV) with four samples. A 470 nm LED (LCS-0470-50-22, Mightex Systems, CA, United States) was used as the light source.

### Data Analysis

The axon detection procedure consisted of three steps: reference spike detection, data extraction, and axon detection (Supplementary Figure 1). First, the signals of the reference electrodes that recorded signals with large amplitudes were subjected to a band-pass filter of 300–3,000 Hz, and negative peaks with absolute values >5 times the standard deviation (SD) of each electrode were identified. When multiple spikes were detected within 50 ms, these spikes were discarded to prevent over-detection of the same spike. To separate signals from multiple neurons, a scatter plot of spike times and amplitudes was created, and the signals were manually sorted into clusters based on their amplitude values.

The signals from all electrodes were then filtered using a band-pass filter at 100–3,000 Hz, and data obtained from −5 to 15 ms were extracted at each spike (Supplementary Figure 1E). When spike triggered averaging is performed, the extracted data contains a mixture of highly reproducible axonal signals and those from surrounding unrelated cells. The signal from the cell body is approximately 100 times larger than that from the axon; thus, the signal-to-noise ratio is reduced when it is mixed. Therefore, for each electrode, the mean and SD of each measurement point in the extracted data were calculated, and signals that exceeded two times the SD and had a peak of > 50 μV were regarded as noise and excluded from the analysis. The signals were averaged for each electrode. Electrodes with < 20 spikes were excluded from the analysis.

Negative spikes were detected in two-dimensional (2D) data at each time point (Supplementary Figure 1F) after filtering with median and Gaussian filters. We used a 2D image peak finder algorithm [Fast 2D peak finder^1^; MATLAB Central File Exchange (Retrieved April 6, 2021)] for peak detection. Spikes within a distance of 87.5 μm (five electrodes) and a time-difference of 50 μs (a sampling point) were combined and considered as arising from the same axon segment (Supplementary Figure 1G). Furthermore, when the ends of the fragments were within 175 μm (ten electrodes) in distance and 400 μs (eight sampling points) in time-difference, they were combined (Supplementary Figure 1H). Finally, axon segments > 300 μm were detected.

For evaluating the background noise level, electrodes with the largest signal of < 30 μV were selected, and the mean value was calculated at each neuron. For evaluating the effect of outlier removal, electrodes under the cell body were selected with an amplitude threshold of 30 μV from the result of activity scan, and the mean value at each neuron was calculated.

To evaluate spatial and temporal characteristics of fast conducting segments, the segments with a conduction velocity of > 1.5 m/s were extracted. To evaluate the spatial characteristics of conduction, we performed current source density (CSD) analysis. CSD data were generated from 2D data at each timepoint using methods from a previous study (Leski et al., 2011). The minimum value of CSD data during the conduction were calculated as maximum sink values at each electrode. Ratios of spike amplitudes and sink values between the edge and center of the segments were calculated.

### Immunocytochemistry

Cells were fixed using 4% paraformaldehyde (PFA; FUJIFILM Wako Pure Chemical Corp.) at room temperature for 30 min on 29th DIV for culture dishes and 77th DIV for HD-MEAs. After rinsing the samples with phosphate buffered saline (PBS, Thermo Fisher Scientific), 4% Block ace (Sumitomo Dainippon Pharma, Osaka, Japan) and 0.25% Triton X-100 (Merck, Darmstadt, Germany) diluted in PBS were added to the sample and left overnight at 4°C. Primary antibodies were diluted with 0.4% Block ace and 0.25% Triton X-100 in PBS, added to the samples, and left at 4°C for 2–3 days. After rinsing with PBS three times for 5 min, the secondary antibody solution was added to the samples and incubated at 4°C for 8 h. After washing with PBS, the samples were observed under a fluorescence microscope. For observing cells on HD-MEAs, the sample was mounted with ProLong Gold Antifade Mountant with DAPI (Thermo Fisher) and clear seal for multi-well plates. The length of the myelin sheath was measured at 219 locations from three images. The following primary and secondary antibodies were used: anti-beta 3 tubulin (mouse, 1:500; Sigma-Aldrich), anti-myelin basic protein (anti-MBP, rabbit, 1:500; Abcam, Cambridge, United Kingdom), anti-pan Nav (mouse, 1:1,000; Abcam), anti-Caspr (rabbit, 1:500; Abcam), anti-P0 (rabbit, 1:1,000, Abcam), Alexa Fluor 488 anti-mouse IgG (goat, 1:500; Thermo Fisher Scientific), and Alexa Fluor 546 anti-rabbit IgG (goat, 1:500; Thermo Fisher Scientific).

For quantitative analysis of myelin segments, a region of interest (ROI) with the size of 250 pixel by 250 pixels was manually selected from each image. Total axon length was measured with NeuronJ, an ImageJ plugin. MBP-positive segments were counted, and normalized with the total axon length. The colocalization ratios of Nav and Caspr were calculated from three independent area with merged images of Nav and Caspr.

### Transmission Electron Microscopy Observation

On the 29th and 70th DIVs, the samples were fixed with 2% PFA and 2% glutaraldehyde in 0.1 M cacodylate buffer pH 7.4 at 4°C. Thereafter, they were fixed with 2% glutaraldehyde at 4°C overnight. Next, the samples were rinsed 3 times with 0.1 M cacodylate buffer for 30 min each and were then fixed with 2% osmium tetroxide at 4°C for 1 h. The samples were dehydrated in graded ethanol solutions (50%, 70%, 90%, anhydrous). The samples were transferred to a resin (Quetol-812; Nisshin EM Co., Tokyo, Japan) and polymerized at 60°C for 48 h. The polymerized resins were ultra-thin sectioned at 70 nm with a diamond knife using an ultramicrotome (Ultracut UCT; Leica, Vienna, Austria), and the sections were mounted on copper grids. They were stained with 2% uranyl acetate at room temperature for 15 min, and washed with distilled water, followed by secondary staining with a lead stain solution (Sigma-Aldrich) at room temperature for 3 min. The grids were observed using a TEM (JEM-1400Plus; JEOL Ltd., Tokyo, Japan) at an acceleration voltage of 100 kV.

### Simulation

In the case of an ideal myelinated axon, most of the axons would be insulated, and because the node of Ranvier is a small structure and the ion current across the axon membrane is also small, the amplitude could clearly decrease depending on the distance between the axon and the electrode. Numerical simulations were performed to show the distance-dependent amplitude variation. To evaluate conduction velocity along axons with myelin sheath, we adapted the mathematical model of action potential propagation along myelinated axons (Richardson et al., 2000). The model implemented in MATLAB (MathWorks) environment (Arancibia-Cárcamo et al., 2017) was downloaded from GitHub^2^. Parameters shown in Supplementary Table 1 were adapted from previous studies (Arancibia-Cárcamo et al., 2017; Cullen et al., 2021) and from experimental data.

The model has 50 nodes and 49 internodes and was expressed as an equivalent circuit (Supplementary Figure 3). The nodes consisted of one compartment and the internode consisted of 82 compartments that were 1 μm long. Each node has three types of active channels: fast and persistent Na channels and a slow K channel, which follow the Hodgkin–Huxley type equations (Richardson et al., 2000). Both nodes and internodes have passive membrane mechanisms: membrane conductance and capacitance. We calculated the intracellular potentials of the axon and of the peri-axonal space, the space between the axon and the myelin sheath, in each compartment for each time in 1 μs steps after an initial current stimulus (amplitude, 5 nA; duration, 5 ns) at time 0. We ran the simulation in 1 μs steps for 3 ms.

The extracellular potential measured by HD-MEA was estimated from the ionic current flowing through the cell membrane of the axon, assuming that one electrode was 7 μm × 7 μm and placed with 11 μm interval between electrodes. The signals of 29 × 9 electrodes were calculated. The axon was assumed to be parallel to the horizontal axis, *d* μm away from the central electrode, and *h* μm in height. The electrical conductivity of the extracellular space is assumed to be isotropic. After calculating the extracellular potential at each point on a 1 μm mesh, the signals at the electrode regions were averaged to simulate the signals measured at the electrodes. Amplitude ratios were calculated for the extracellular potential data and for the filtered signal at the electrodes. Calculations were performed at different distances and heights from the electrodes to evaluate the effect of the spatial arrangement of the electrodes and axons on the signals.

### Statistical Analysis

Data are reported as mean ± standard deviation. Statistical analysis was performed with Student’s *t* test with a Bonferroni correction for TEM results and with the paired *t* test with a Bonferroni correction for noise level comparison. Differences were considered significant if *p* < 0.05. For statistical analysis, MATLAB R2019a was used.

### Code Accessibility

The data analysis and simulation were performed using MATLAB running on Windows 10. All data and code are provided upon reasonable request to the corresponding authors.

## Results

### Microscopic Observation of *in vitro* Myelination in Sensory Axons

**FIGURE 1:**
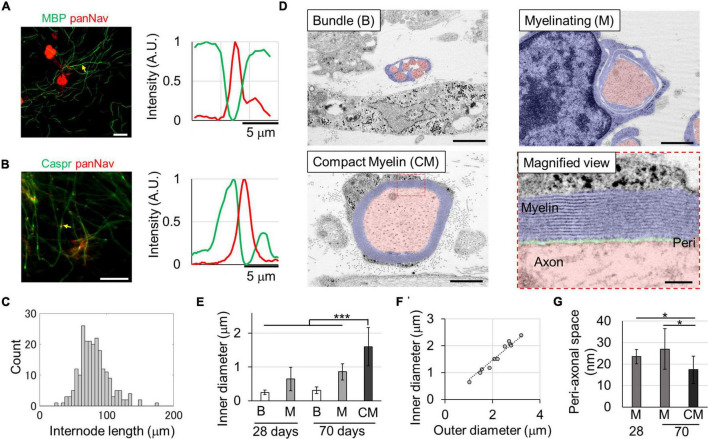
*In vitro* myelin sheath formation. Myelin sheath **(A)** and paranode formation **(B)** at 29 days *in vitro* (DIVs). Relative fluorescence intensities around the yellow arrows are shown in right panels. The myelin basic protein (MBP) or Caspr and panNav protein were alternatively expressed along axons. Heminodes with a single Caspr spot and one panNav spot were also observed. Histogram of the internode length **(C)**. Ultra-structures of the myelin sheath **(D)**. Fully myelinated axons with compact myelin (CM) were observed at the 70th DIV. The diameter of myelinated axons was significantly larger than that of other axons **(E)**. The g-ratio, which is the ratio between inner and outer diameters, was 0.74 **(F)** (R^2^ = 0.896, *p* = 3.1 × 10^– 13^). The peri-axonal space indicated in green in the magnified image was smaller in myelinated axons than in wrapped axons **(G)**. *p < 0.05, ***p < 0.001; Student’s *t* test. Error bars indicate standard deviation. Scale bars, 50 μm in panels **(A,B)**; 1 μm in panel **(D)**; 100 nm in panel **(D)** magnified view.

To confirm the formation of the myelin sheath in the culture environment, rat DRG cells were isolated and cultured. The results of immunostaining on the 29th DIV are shown in Figure 1. The expression of MBP, myelin sheath markers, and small clusters of voltage-dependent Na channels (Navs), Na channel markers, was observed. MBP-positive structures and Nav-positive spots were observed alternately (Figure 1A), indicating the formation of the myelin sheath and node structures. The MBP-positive segments was observed with the density of 4.4 ± 2.6 segments/mm. To confirm the formation of paranodes, we assessed the expression of Caspr, a paranode marker, and Nav. Nav-positive spots were observed between two Caspr-positive spots (Figure 1A, arrows; 13%), and in major cases, one Caspr-positive spot was found adjacent to a Nav-positive spot (Figure 1A, arrowheads; 87%), suggesting that Nav was still localized in the heminode. The length of the MBP-positive structure was 81.8 ± 22.4 μm (Figure 1C).

To evaluate the myelin sheath in greater detail, the ultrastructure was observed using TEM. On the 28th DIV, unmyelinated axons bundled by single Schwann cells and axons wrapped with Schwann cells in the process of forming myelin sheaths were observed (Figure 1D), but very few compact myelin sheaths were observed. However, compact myelin was observed on the 70th DIV as similar frequency as developing myelin sheaths. The diameter of the axons in the different stages of myelination was compared (Figure 1F), and there was a significant difference between them [Compact myelin (CM) vs. Myelinating (M) on the 70th DIV, *p* < 0.01; M vs. the other, W vs. the bundle (B), *p* < 0.001]. The diameter range was 0.13–0.58, 0.21–1.28, and 0.64–2.40 μm for bundled, myelinating, and myelinated axons, respectively. Additionally, the diameter of myelinating axons was significantly greater on the 70th DIV than on the 28th DIV (*p* < 0.01), suggesting that axons grew with the passage of time.

Paranode structures were also observed on the 70th DIV. The interval between the myelin membranes was 15.9 ± 0.9 nm, and the myelin sheaths wrapped around the axons an average of 14.8 ± 4.3 times. The g-ratio, which is ratio of the outer diameter (including the myelin sheath) to the axon inner diameter, was 0.74 (Figure 1F: linear regression, R^2^ = 0.896, *p* = 3.1 × 10^–13^), which is in the range of the optimum values observed in theoretical studies (0.6–0.77) and in experimental results of previous studies (Chomiak and Hu, 2009; Cohen et al., 2020). Additionally, the peri-axonal space was measured in wrapped and myelinated axons (Figure 1G). The peri-axonal space was narrower in myelinated axons than in wrapped axons, suggesting that the peri-axonal space becomes narrower with myelin formation. These results indicate that myelin sheaths are still in the development process when cultured for approximately 1 month, and that compact myelin sheaths are completed when cultured for up to 70 days.

### Recording Action Potential Propagation Along Sensory Axons

**FIGURE 2:**
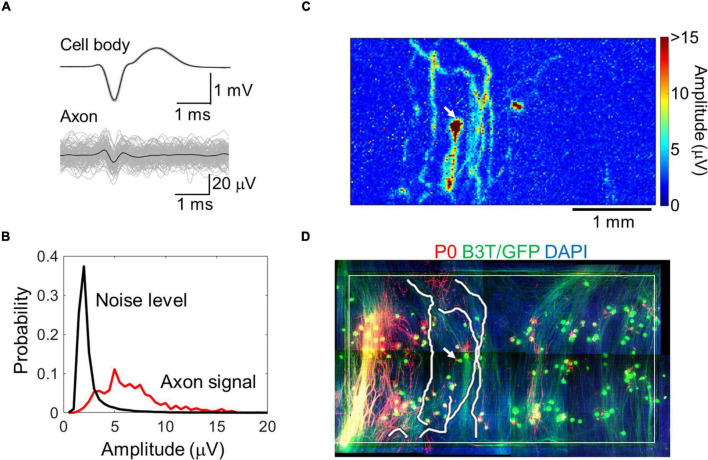
Axon signal detection with a high-density microelectrode array. Averaged cell body and axon signals (black) with individual signals (light gray) **(A)**. Comparison between the axon signal and noise level **(B)**. Spike amplitude map for a representative neuron calculated with the spike triggered averaging method **(C)**. Detected axon segments and immunofluorescent image of the neuron **(D)**. Red, green, and blue colors show P0 protein (myelin sheath), green fluorescent protein and beta III tubulin (neuron), and DAPI (cell nuclei), respectively. The white rectangle shows the recording area. Image for the whole recording area was made by combining multiple images. Electrical recording at 58 DIV, immunofluorescent imaging at 77 DIV.

By spike-triggered averaging, the background noise level effectively decreased (Supplementary Figure 2). In subsequent analyses, we averaged over 20 spikes for each electrode. Electrodes with insufficient number of spikes were excluded from the analysis. The signal-to-noise ratio improved, and the signals from axons with smaller amplitudes (approximately 5 μV) could be better detected (Figures 2A,B and Supplementary Movie 1). A map of the spike amplitudes is shown in Figure 2C. Axon arbors with relatively large amplitudes were detected. The axonal signals were detected in all samples. Detected axon arbors of single neurons were stacked within the immunofluorescent image (Figure 2D and Supplementary Movie 2), showing that the positions of detected axons were generally consistent with those of B3T-positive axon bundles. We detected 13 ± 8 neurons from a chip, and each neuron had 6.8 ± 4.1 axon segments. The total axon length per neuron was 7.1 ± 4.5 mm on 58–64 DIV.

**FIGURE 3:**
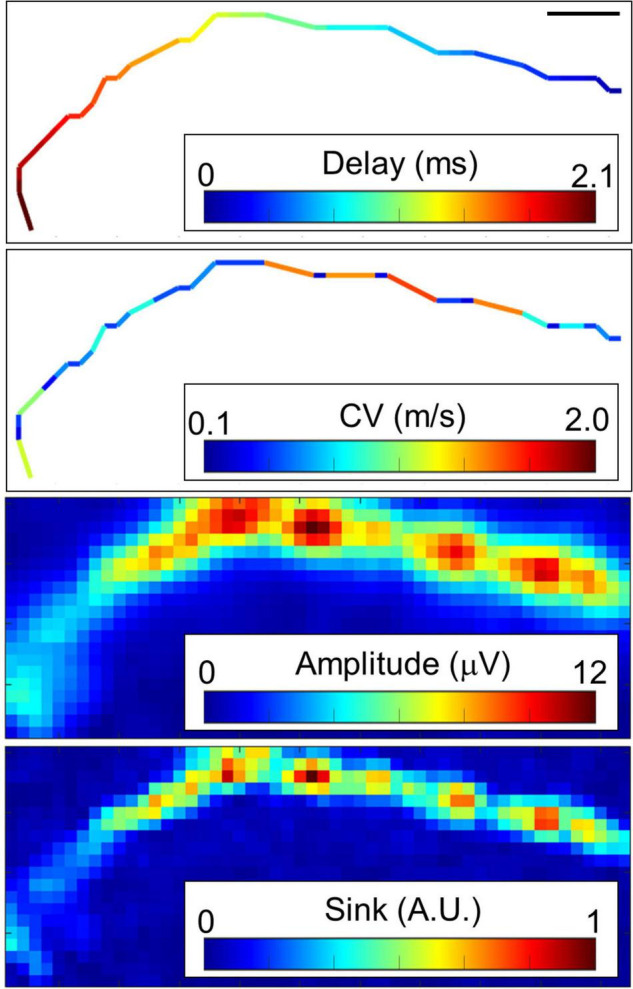
Representative propagating signals along axons. Propagating signals in some axons show variant conduction velocity (CV). Amplitude and electrical sink calculated with current source density analysis also show variation along the axon. Note that the CV was inclined to be lower in the locations displaying higher amplitudes and electrical sink. The scale bar indicates 100 μm. More than 20 spikes were averaged at each electrode. Electrical recording at 61 DIV.

Typical conductions are shown in Figure 3 and Supplementary Figure 4. When myelinated axons are formed, Na channels are concentrated at the node of Ranvier, and the electrical sink, which indicates the amount of influx of Na ions, is expected to be locally large. Therefore, we calculated the electrical sink from the 2D amplitude data by CSD. Since the amplitude of the extracellular potential varies due to the local filtering effect of the cells and electrodes, the electrical sink was normalized to the maximum value in the image. Some axons showed segments with variable conduction velocity (Figure 3). The conduction included low amplitude segments, and the conduction velocity was inclined to be lower in the locations displaying higher amplitudes and electrical sink. At both ends of the fast-conducting segments, the conduction velocity tended to be low while the amplitude was high.

**FIGURE 4:**
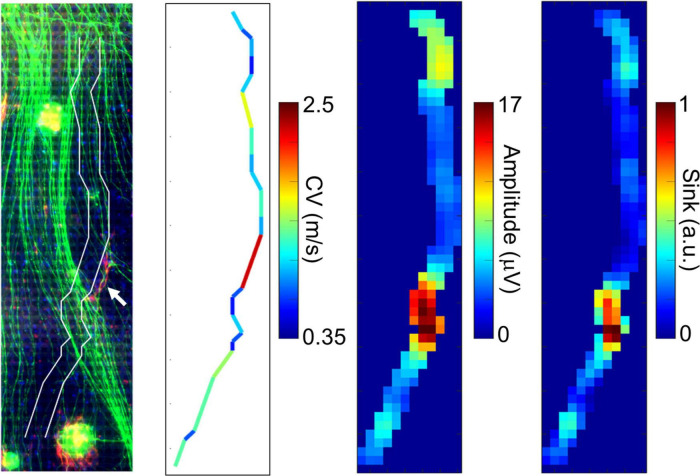
Comparison between immunofluorescent image and fast conducting segment. The detected axon segment is stacked on the immunofluorescent image using antibodies to beta III tubulin (green) and P0 protein (red). The axon segment is positioned between two white lines. The white arrow shows the position of P0-positive segment. The fast-conducting segment, low amplitude, and sink position were located in vicinity of the P0-positive segment. More than 20 spikes were averaged at each electrode. Electrical recording at 61 DIV, immunofluorescent imaging at 77 DIV.

We compared the electrically detected axons and magnified immunofluorescent images in order to examine if the fast-conducting segment was located in vicinity of P0-positive myelin sheathes. A representative segment is shown in Figure 4. Although the position is tens of micrometers apart, the position of the axon segment is consistent with that of P0-positive segment showing myelin sheath (white arrow in Figure 4, left panel), and low amplitude and sink area. The result suggests that the fast-conducting segment can include saltatory conduction along myelinated axon.

### Spatial and Temporal Properties of Fast-Conducting Segments

**FIGURE 5:**
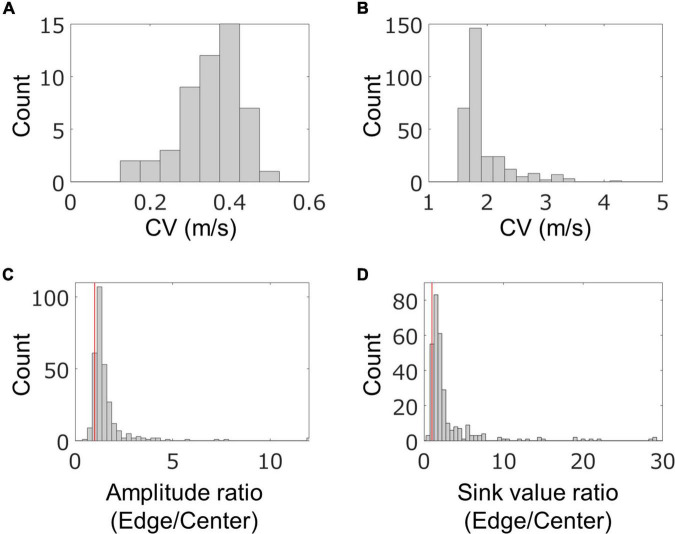
Spatial and temporal characteristics of fast conduction segments. The histograms show conduction velocity (CV) for entire axons **(A)** and fast conduction segments **(B)**. Fast conduction segments showed 5.5 times faster conduction. The ratio of the amplitudes **(C)** and electrical sink **(D)** between the center and the edge in the fast segment was calculated for evaluating spatial properties. More than 90% of fast conduction segments showed greater value for amplitude and sink at the edge than the center. Red vertical lines are placed at 1 in panels **(B,C)**. Electrical recording on 58–64 DIV.

In order to quantitatively evaluate the characteristics of the fast conduction segments, we extracted the segments from the four samples on 58–64 DIV and evaluated their spatial and temporal characteristics. The results are shown in Figure 5. In one chip, 83 ± 58 segments were detected from 91.5% of neurons, and their density was 1.1 ± 0.8 segments/mm. Their length was 96.6 ± 21.6 μm, which was consistent with the results of immunofluorescent imaging. The mean conduction velocity along entire axons was 0.35 ± 0.08 m/s (Figure 5A), which is similar to the conduction velocity in unmyelinated axons in previous studies (Raastad and Shepherd, 2003; Bakkum et al., 2013; Shimba et al., 2015; Bonalume et al., 2021). The conduction velocity of the fast conduction segment was 1.9 ± 0.4 m/s (Figure 5B), which is 5.5 times faster than entire axons. The ratio of the amplitudes of the center and the edge of the fast segment was calculated to be 1.57 ± 1.13, and 90.7% was greater than 1 (the red line in Figure 5C is placed at 1). Similarly, the sink ratio was calculated to be 3.12 ± 4.25, and 92.8% was greater than 1 (Figure 5D). Similar tendency was observed with the samples at 35 DIV as shown in Supplementary Figure 5. These results indicate that the fast conduction segment is comparable to the length of the myelin sheath obtained by immunofluorescent staining and is faster than the entire axon. The amplitude and sink were larger at the ends than at the center.

### Simulation of Extracellular Potential Along Myelinated Axons

**FIGURE 6:**
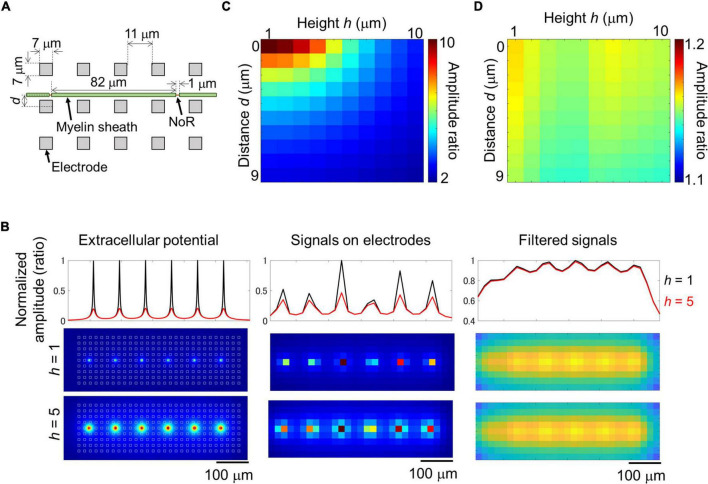
Simulation of extracellular potential along myelinated axon. Structural parameters of the simulated myelinated axon and electrodes **(A)**. Maximum amplitude of extracellular potential around the myelinated axon at 1 μm mesh [**(B)** left panels], and on electrodes [**(B)** center and right panels]. Recorded signal was simulated with 2D median and Gaussian filters (right panels, filtered signal). White squares in panel **(B)** left panel shows the position of electrodes. Black and red lines show normalized amplitude at the heights of 1 and 5 μm. As the height between axon and electrodes increased, the amplitude at the node of Ranvier (NoR) decreased [**(B)** top panels]. Relationship between the distance and height and amplitude ratio calculated from the amplitude of electrodes under NoR and myelin sheath [**(C)** unfiltered; **(D)** filtered]. Note that the distance caused few effects on the amplitude ratio of the filtered signal as shown in panel **(D)**.

Results of imaging and electrical recording show that we probably recorded signal from small nodes of Ranvier in myelinated axon with larger electrodes. In the case of an ideal myelinated axon, most of the axons would be insulated, and because the node of Ranvier is a small structure and the ion current across the axon membrane is also small, the amplitude could clearly decrease depending on the distance between the axon and the electrode. It is not clear if such large electrodes can effectively record signal from small targets. We simulated the extracellular potential recorded from myelinated axons. A mathematical model was created based on the results of the imaging experiments and electrode size as shown in Figure 6A. The extracellular potential around the myelinated axon was calculated (Figure 6B left), and then those on electrodes were calculated with and without 2D filtering which is same to the electrical recording experiment (Supplementary Movie 3; without filtering, Figure 6B center and Supplementary Figure 6A; with filtering, Figure 6B right, and Supplementary Figure 6B). As the distance between electrodes and axon increased, the amplitude ratio of unfiltered signal between under the node of Ranvier and under the myelin sheath decreased from approximately 10 to 2 (Figure 6C). Meanwhile, the amplitude ratio of filtered signal did not show clear differences among different distance between electrodes and axon (1.1–1.2; Figure 6D), which is similar value to the ratio in the experiment. These results show that extracellular potential of myelinated axons can be recorded from electrodes on the HD-MEA, and the position of the node of Ranvier can be detected by the amplitude.

## Discussion

In this study, we aimed to detect saltatory conduction in peripheral neurons using HD-MEAs. Fluorescent imaging and TEM examination showed that the myelin sheath was formed in the culture condition. The activity of DRG neurons was measured in the HD-MEA, and axon conduction with a velocity of more than 2 m/s was observed. The amplitude and electrical sink at ends of the fast-conduction segments were higher than those at the center of the segments. Furthermore, a mathematical model based on the TEM and immunofluorescent staining results showed that saltatory conduction along myelinated axons can be recorded with HD-MEAs. Overall, these results showed that saltatory conduction was recorded with HD-MEAs.

The fast conduction segments with velocity of > 2 m/s and large electrical sink and amplitude at the ends were detected. This is because either part of the conduction in the unmyelinated axon was undetected, or it was saltatory conduction along myelinated axons including incompletely myelinated ones. The first possibility that it represented conduction in an unmyelinated axon is unlikely for the following reason. Numerical calculations show that conduction velocity of 2 m/s occurs in unmyelinated axons with diameters of approximately 20 μm (Ashida and Nogueira, 2018). Contrastingly, the diameter of unmyelinated axons was around 0.3 μm, and the maximum diameter of unmyelinated axons was 0.58 μm, as noted from TEM observations. Therefore, the conduction velocity observed here is unlikely to occur in unmyelinated axons. Therefore, it is likely that the fast conduction segment detected here utilized saltatory conduction.

TEM showed that the formation of the myelin sheath was incomplete at 29th DIV, and only myelinating axons were observed. Clustering of Na channels and localization of Caspr in the heminode were observed in the immunofluorescence study. Because two heminodes fuse and form the complete node (Freeman et al., 2015, 2016), the results suggest the existence of a developing myelin sheath at this point in time. The results are consistent with those of previous studies, which showed that developing myelin sheath and the formation of Na channel clusters is sufficient to increase the conduction velocity (Freeman et al., 2015).

Although some fast conduction segments showed corresponding P0-positive segments indicating myelin sheath in the immunofluorescent image, many of them did not. This may be due to the incomplete myelin sheath and the setup of the equipment. In this study, activity recordings were performed at 9 WIV. Results of TEM imaging suggest the existence of immature myelin sheath at that time point. Thus, it is possible that the myelin sheath was not formed until the P0 protein was sufficiently observed. The electrical data at 35 DIV also suggest that developing myelin sheath is sufficient for saltatory conduction. It may be effective to use culture conditions that facilitate myelin sheath formation (Bacallao and Monje, 2015) and to extend the culture period for measurement. In addition, a low-magnification objective lens was used to avoid interference with the culture ring of the chip. In the future, using chips with a larger culture ring allows use of a higher magnification objective lens, and developing myelin segments with thin structure and low fluorescent intensity can be detected.

Simulated extracellular potentials for the myelinated axon suggest that activity can be measured with electrodes that are larger in size than the node of Ranvier. In this study, two-dimensional filters were applied to the two-dimensional extracellular potential data for reliable detection of small axonal signal. Simulation results showed that the ratio of amplitude and sink became robust to the distance between the electrode and axon after filtering, while the ratio decreased. Since the decrease in the ratio may make the detection of nodes more difficult, it may be effective to apply more sophisticated image processing techniques instead of filtering. In addition, there was a segment with a higher ratio than the theoretical value. This may be because a large signal was measured from the node because of glial cells covering the node (Matsumura et al., 2016), or smaller signals were measured due to cells existing between the myelin sheath and the electrode.

The reason why saltatory conduction could be recorded in this study is as follows. First, the formation of myelination was limited as shown by immunostaining. Thus, the spike triggered averaging worked well to detect axons, as previous studies did to unmyelinated axons (Bakkum et al., 2013). Second, by smoothing the two-dimensional images, the intermittent signals from the nodes of Ranvier were connected and could be detected as continuous signals from the whole axon.

This method has two major advantages over conventional methods: first, it can measure the activity of multiple axons in a wide area simultaneously. In the VSD and patch-clamp methods, the activity is measured from one axon at a time. The VSD acquires images usually from an area of 2–6 μm^2^, and the loading time of the dye is 2–3 h per axon (Cohen et al., 2020). The patch-clamp method can record activity from several points where electrodes are attached, and previous studies have shown that skilled researchers can measure from approximately four axons per day (Sasaki et al., 2012). Here, we could acquire signals from more than 10 neurons, including an axonal length of 2 mm per neuron in a single measurement (20 min for activity scanning and 45 min for axonal recording). Thus, this method can be used for evaluating axon characteristics on a specific part of the axons because of its wide range of measurement and high throughput.

The second advantage is the suitability for long-term measurement. Conventional methods are invasive and can lead to cell damage, and the measurement time using the same for a single axon is only a few hours (Emmenegger et al., 2019). However, our method does not damage cells and they can be used for measurements over several days. Therefore, this method can be used for the long-term evaluation of phenomena such as demyelination and remyelination, which occur over several days.

The limitation of the present method is that it is difficult to evaluate rare phenomena and transient changes because the axonal signal was detected by averaging multiple signals. In a previous study, a similar device was used to detect single firings in axons by template matching and local correlation (Radivojevic et al., 2017; Bullmann et al., 2019). Here, we believe that each spike can be detected using the same operation. Moreover, additional control experiments with samples on earlier time points or with cell division inhibitor will further support our results.

We have succeeded in electrically measuring saltatory conduction with spatial information using HD-MEAs for the first time. Our method provides long-term and high throughput recordings, which can help evaluate the process of demyelination and remyelination in the long run, and a platform for screening new compounds for demyelination diseases.

## Data Availability Statement

The raw data supporting the conclusions of this article will be made available by the authors, without undue reservation.

## Ethics Statement

The animal study was reviewed and approved by the University of Tokyo Animal Experiment Committee.

## Author Contributions

KeS: conceptualization, methodology, investigation, and writing—original draft. TA: software and writing—review and editing. KoS: methodology and writing—review and editing. KK: validation and writing—review and editing. YJ: supervision and writing—review and editing. All authors contributed to the article and approved the submitted version.

## Conflict of Interest

The authors declare that the research was conducted in the absence of any commercial or financial relationships that could be construed as a potential conflict of interest.

## Publisher’s Note

All claims expressed in this article are solely those of the authors and do not necessarily represent those of their affiliated organizations, or those of the publisher, the editors and the reviewers. Any product that may be evaluated in this article, or claim that may be made by its manufacturer, is not guaranteed or endorsed by the publisher.

## Footnotes

1
https://www.mathworks.com/matlabcentral/fileexchange/37388-fast-2d-peak-finder

2
https://github.com/AttwellLab/MyelinatedAxonModel
