# Supplementary material for: Recording Saltatory Conduction Along Sensory Axons Using a High-Density Microelectrode Array
Source: Front Neurosci. 2022 Apr 18;16:854637. doi: 10.3389/fnins.2022.854637 (PMC9058065; doi:10.3389/fnins.2022.854637)
Supplement: Supplementary file 1 [file Data_Sheet_1.pdf]

Supplementary Material

Recording saltatory conduction along sensory axons using a high-density microelectrode array

1 Supplementary Figures

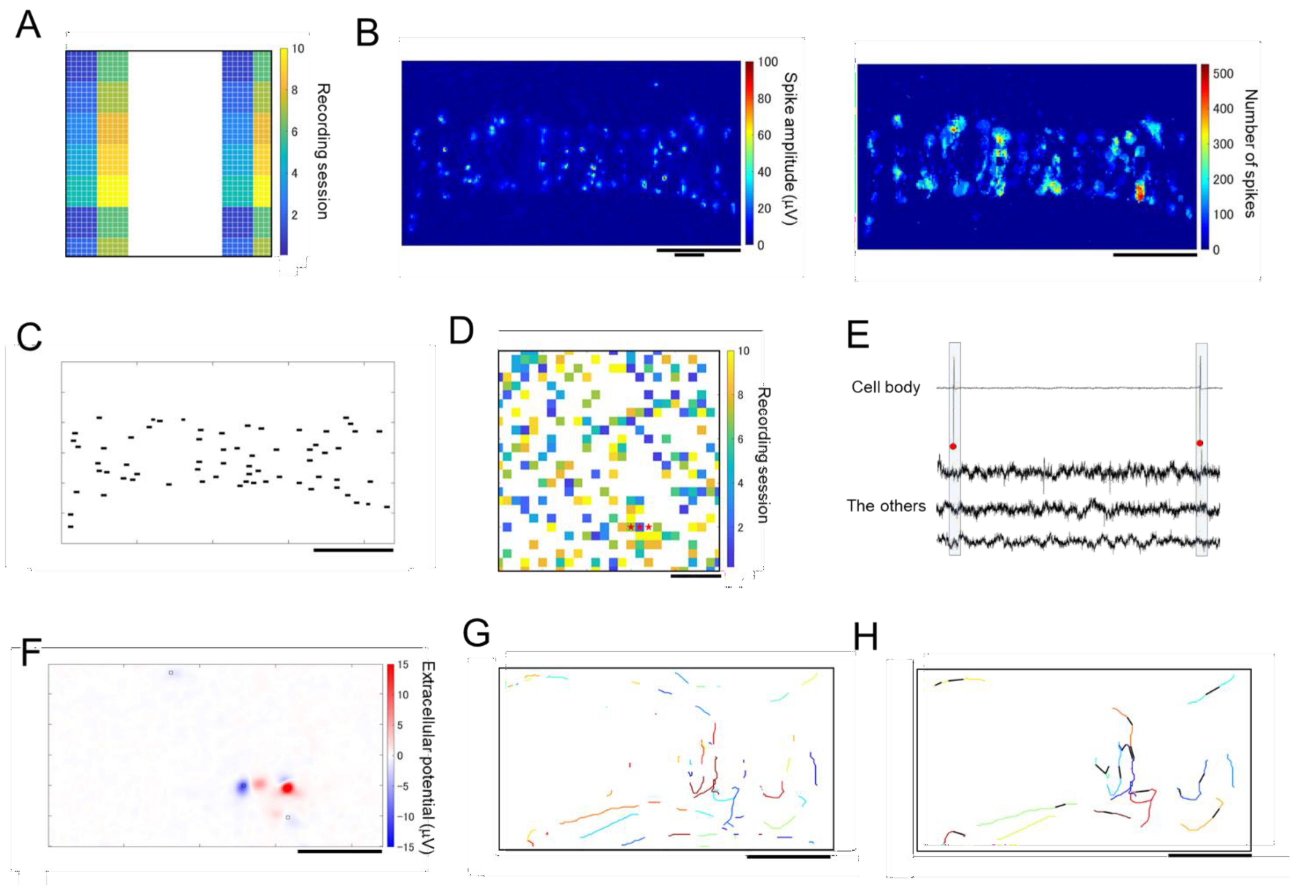

Supplementary Fig. 1. Recording and analysis procedure.

Electrodes were divided into multiple sessions and scanned (A). Spike amplitudes and number of spikes at each electrode were computed (B). Electrodes with a larger amplitude and greater spike number were selected as the cell body electrode (C). The cell body electrodes (red stars) were fixed, and the other electrodes were scanned (D). Spikes of the cell body were detected, and signal around each spike was extracted (E). From the extracted signal, negative peaks on the extracellular map were detected (F). Detected peaks were combined (G), and then these segments were connected (H). Scale bars indicate 100 μm in A and D; 1 mm in the others.

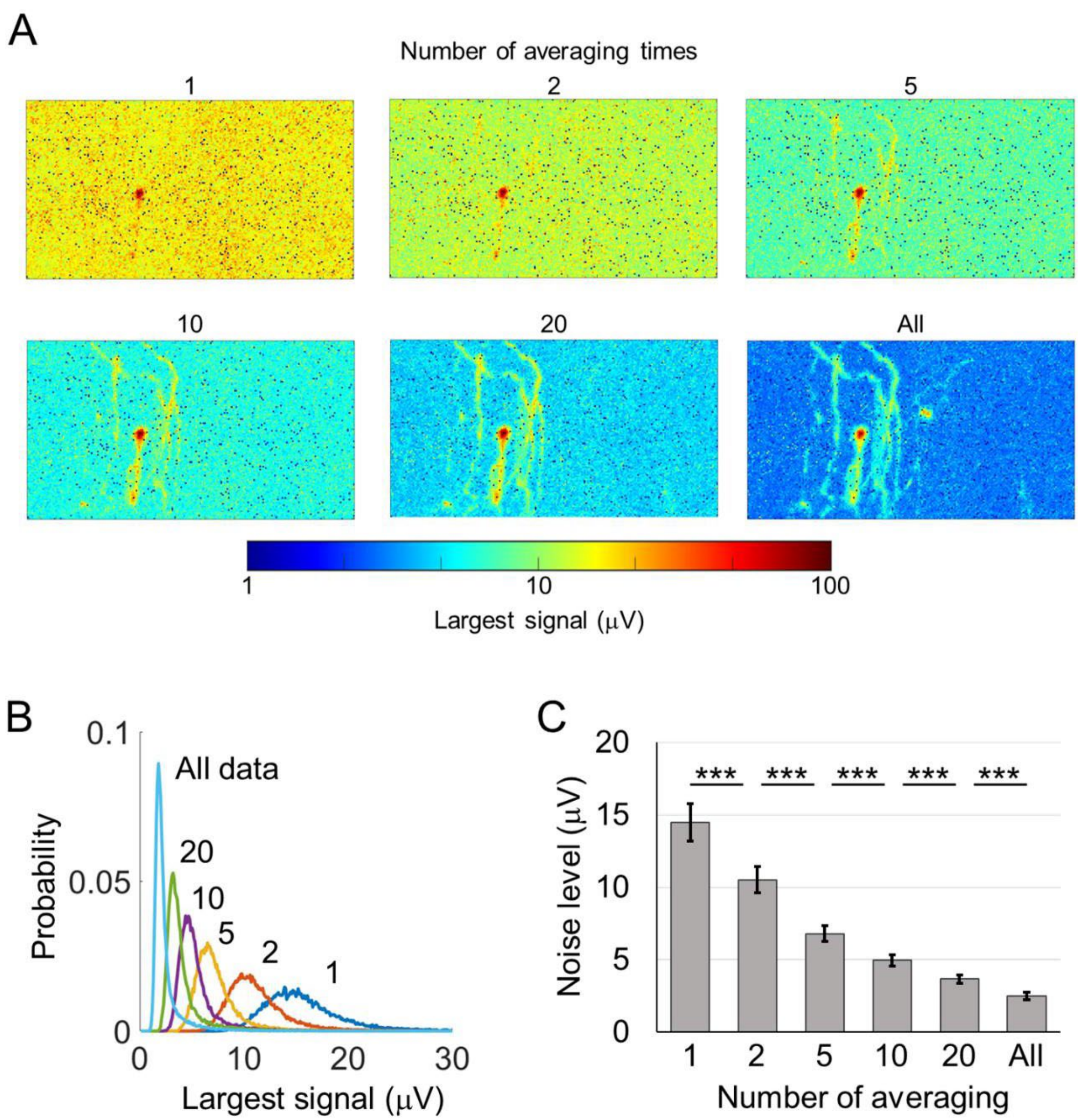

Supplementary Fig. 2. Noise reduction with averaging and outlier removal.

Signal and noise amplitude maps for a representative neuron (A). Histograms for noise level of a representative neuron (B) and all neurons (C) show that the background noise level decreased when the number of averaged data increased. ***, $p < 0.001$; paired t test with Bonferroni correction, n = 51 neurons from 4 chips. Error bars indicate standard deviations.

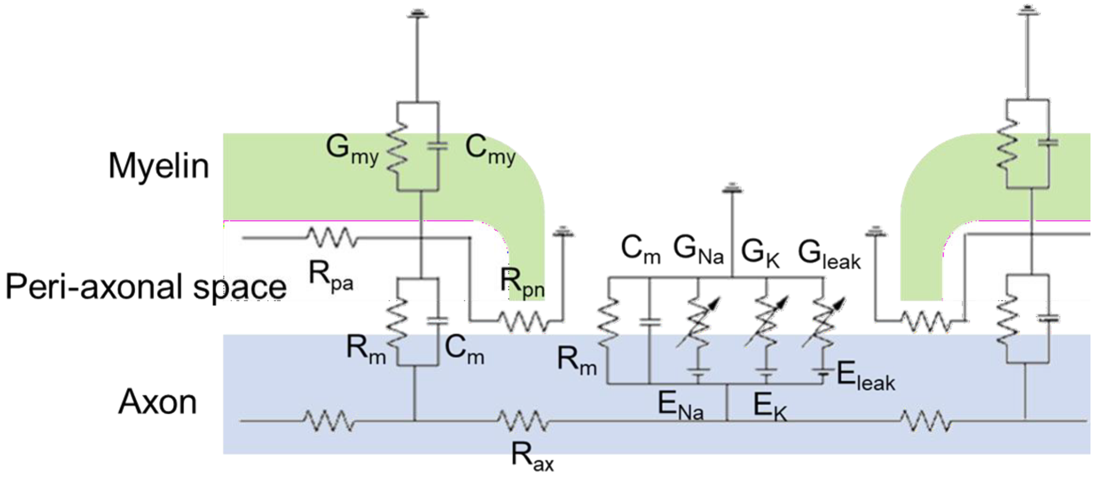

Supplementary Fig. 3. Mathematical simulation of saltatory conduction.

Equivalent circuit model for simulation. All parameters are shown in Supplementary Table 1.

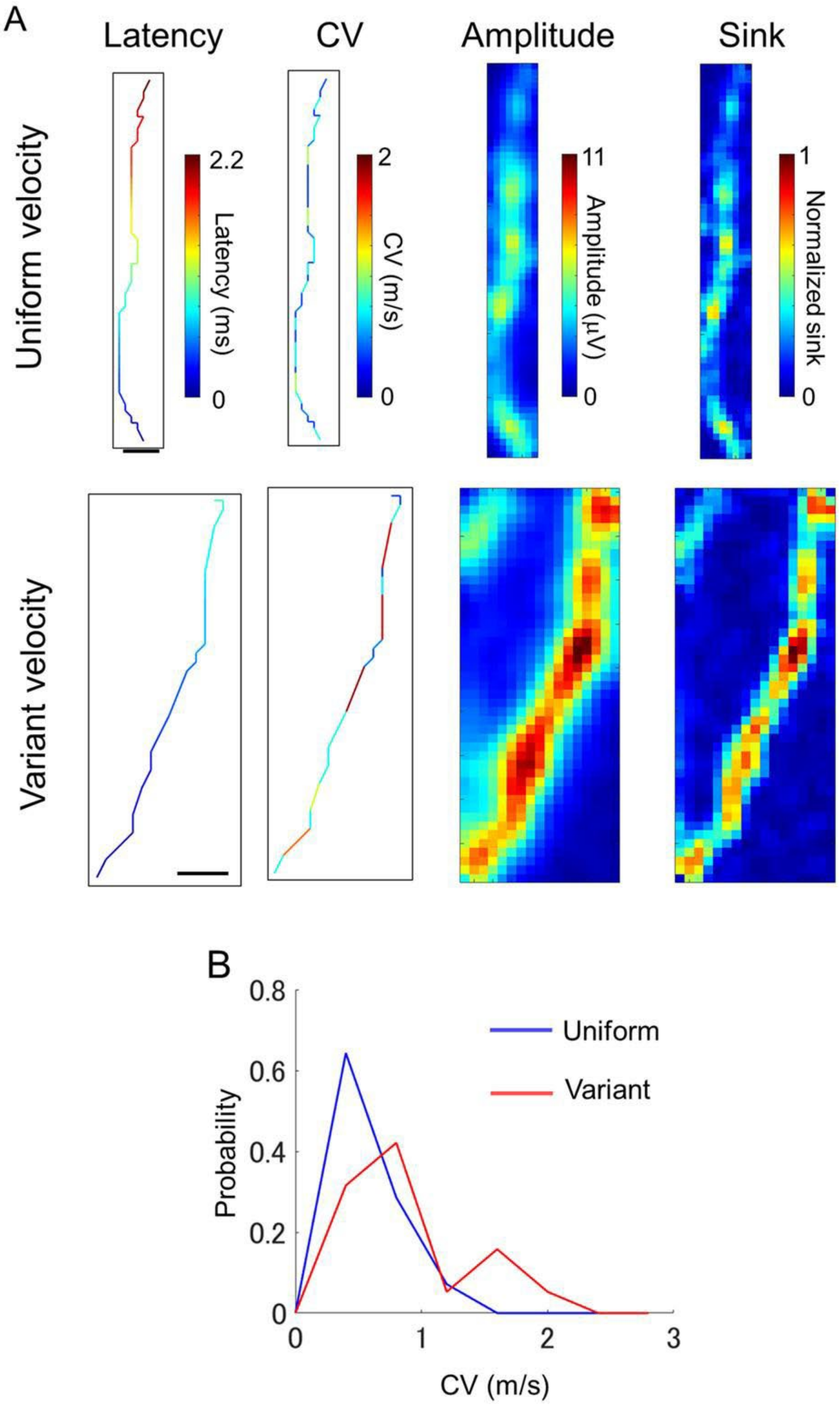

Supplementary Fig. 4. Representative axons with uniform and variant conduction velocity.

(A) Representative electrical profiles along axons with uniform and variant conduction velocity (CV). Scalebars indicate 100 µm. (B) Distribution of CV.

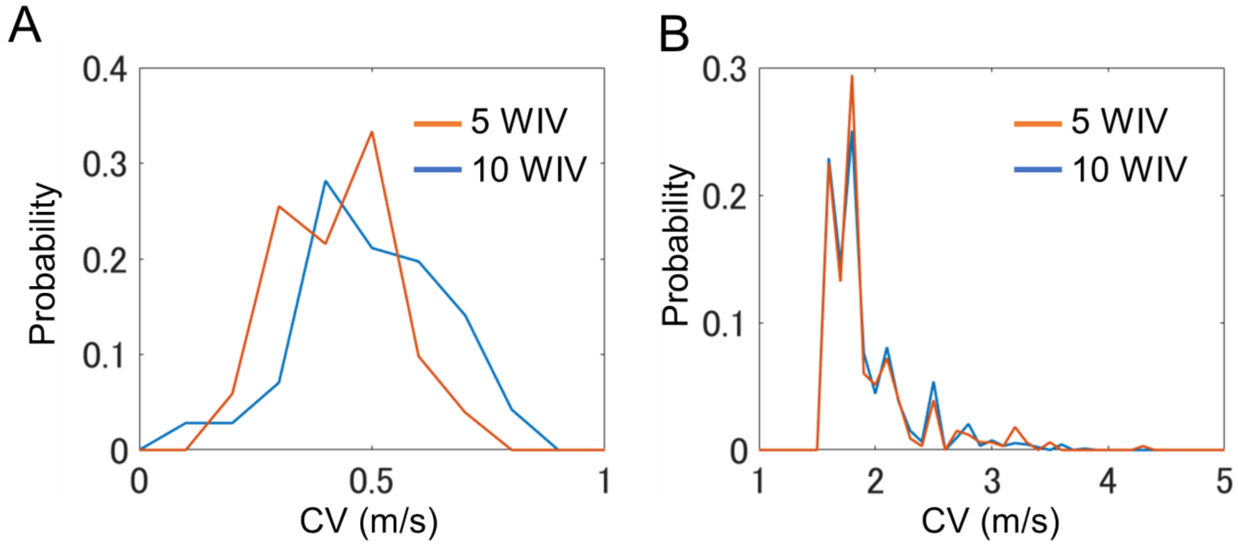

Supplementary Fig. 5. Comparison of conduction velocity between 5 and 10 WIV.

Conduction velocity (CV) was compared between 5 and 10 weeks *in vitro* (WIV). Histograms of averaged CV of neurons (A) and fast conduction segments (B) show similar shapes.

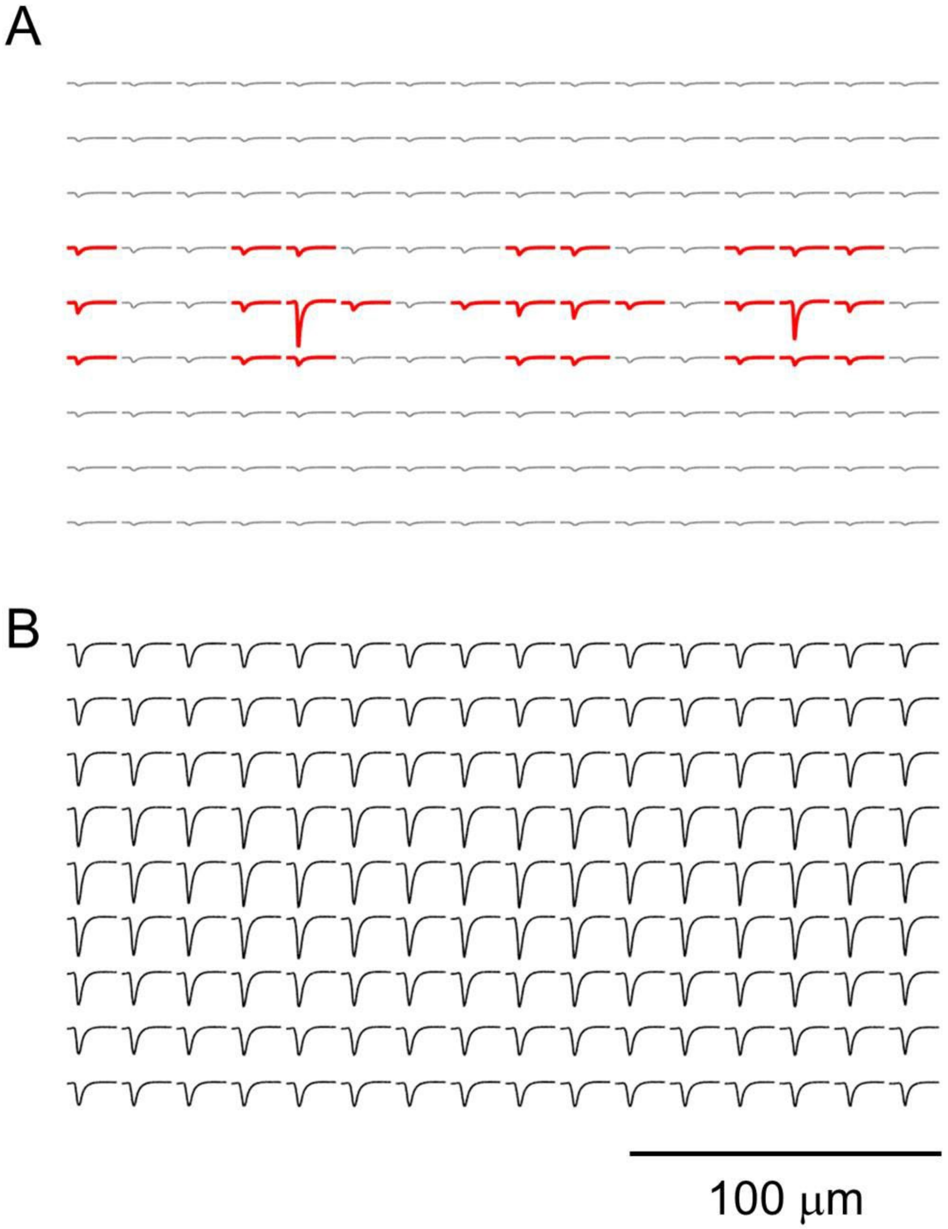

Supplementary Fig. 6. Simulated extracellular potentials at each electrode.

Waveforms of simulated extracellular potentials for 1 ms before (A) and after (B) 2D filtering are plotted. Waveforms in 9×16 electrodes are shown. Waveforms with relatively large signal amplitudes (> 10% of the electrode with the maximum amplitude) are indicated as red in A. After filtering, electrodes near the axon show almost constant amplitude.

2 Supplementary Table

Supplementary Table 1. Parameters for simulation.

Parameter		Value	Units
Temperature		37	°C
Stimulation	Amplitude	5	nA
	Duration	5	nA
Geometrical parameter	Number of nodes	50	
	Number of internodes	49	
	Node length	1	μm
Electrical Parameter	Resting membrane potential	-82	mV
	Leak reversal potential	-83.38	mV
	Axon axial resistivity	0.7	Ω.m
	Membrane capacitance	0.9	μF/cm^2
	Myelin membrane conductance	1	mS/cm^2
	Internode axon membrane conductance	0.1	mS/cm^2
	Peri-axonal space resistivity	0.7	Ω.m
	Nodal Na channel conductance	30	mS/mm^2
	Nodal persistent Na channel conductance	0.05	mS/mm^2
	Nodal K channel conductance	0.8	mS/mm^2
	Na channel reversal potential	50	mV
	K channel reversal potential	-84	mV
Structural parameter	Axon diameter	1.6	μm
	Peri-axonal space width	17.3	nm
	Paranode space width	1	nm
	Myelin wrap periodicity	15.9	nm
	Myelin lamellae	17	times
	G-ratio	0.74	(ratio)
	Internode length	82	μm

3 Captions for Supplementary Movies

Supplementary Movie 1. Extracellular potential landscape of propagating action potential along axon.

Blue and Red colors indicate negative and positive potentials, respectively. The time when the cell body shows action potential was defined as 0.

Supplementary Movie 2. Relationship between detected signals and fluorescent image.

Detected 2D peaks of a representative neuron were plotted on the immunofluorescent image of the same sample. Eight images were combined for showing the whole recording area. Action potential propagated along axon bundle (green, beta III tubulin and green fluorescent proteins). Myelination (red, P0 protein) was observed along axons. Cell nuclei were counter stained with DAPI (blue). Red, yellow, and white dots show the detected peaks at t-0.05 ms, t, and t+0.05 ms (t represents the time indicated in the movie). The time when the cell body shows action potential was defined as 0.

Supplementary Movie 3. Simulated extracellular potential on electrodes.

Extracellular potential along a myelinated axon was simulated. Amplitude was normalized at each condition. Blue and Red colors indicate negative and positive potentials, respectively. The scale bar indicates 100 μm. The h means the distance in height direction between axon and electrode.
